# Language access differentially alters functional connectivity during emotion perception across cultures

**DOI:** 10.3389/fpsyg.2023.1084059

**Published:** 2024-02-15

**Authors:** Joseph Leshin, Maleah J. Carter, Cameron M. Doyle, Kristen A. Lindquist

**Affiliations:** ^1^Department of Psychology and Neuroscience, The University of North Carolina at Chapel Hill, Chapel Hill, NC, United States; ^2^Biomedical Research Imaging Center, School of Medicine, The University of North Carolina at Chapel Hill, Chapel Hill, NC, United States

**Keywords:** emotion, language, culture, concepts, fMRI

## Abstract

**Introduction:**

It is often assumed that the ability to recognize the emotions of others is reflexive and automatic, driven only by observable facial muscle configurations. However, research suggests that accumulated emotion concept knowledge shapes the way people perceive the emotional meaning of others’ facial muscle movements. Cultural upbringing can shape an individual’s concept knowledge, such as expectations about which facial muscle configurations convey anger, disgust, or sadness. Additionally, growing evidence suggests that access to emotion category words, such as “anger,” facilitates access to such emotion concept knowledge and in turn facilitates emotion perception.

**Methods:**

To investigate the impact of cultural influence and emotion concept accessibility on emotion perception, participants from two cultural groups (Chinese and White Americans) completed a functional magnetic resonance imaging scanning session to assess functional connectivity between brain regions during emotion perception. Across four blocks, participants were primed with either English emotion category words (“anger,” “disgust”) or control text (XXXXXX) before viewing images of White American actors posing facial muscle configurations that are stereotypical of anger and disgust in the United States.

**Results:**

We found that when primed with “disgust” versus control text prior to seeing disgusted facial expressions, Chinese participants showed a significant decrease in functional connectivity between a region associated with semantic retrieval (the inferior frontal gyrus) and regions associated with semantic processing, visual perception, and social cognition. Priming the word “anger” did not impact functional connectivity for Chinese participants relative to control text, and priming neither “disgust” nor “anger” impacted functional connectivity for White American participants.

**Discussion:**

These findings provide preliminary evidence that emotion concept accessibility differentially impacts perception based on participants’ cultural background.

## 1 Introduction

Emotion perception—or understanding the emotional meaning of someone else’s facial, body, or vocal behaviors—is crucial to social communication, drives social behavior, and facilitates the social connection that is ultimately critical to human health and wellness (Telzer et al., 2014; Atzil and Gendron, 2017; LoBue et al., 2019; Milojevich et al., 2021). Basic emotion theory classically posits that a set of so-called universal emotions are perceived reflexively in others’ facial configurations based on feature detection alone (Ekman and Friesen, 1971; Scarantino and Griffiths, 2011; Keltner et al., 2019). Yet accumulating evidence suggests that emotion perception depends on conceptual knowledge about emotion that is activated in the minds of perceivers (Hassin et al., 2013; Lindquist and Gendron, 2013; Barrett et al., 2019). This conceptual knowledge is influenced by a person’s prior experiences (e.g., Halberstadt et al., 2009), including their cultural background (Jack et al., 2016; Gendron et al., 2020; Lindquist et al., 2022). Moreover, growing evidence suggests that immigrants’ exposure to a host culture influences their conceptual knowledge about emotions (Gendron et al., 2020) that informs the experience of emotion (De Leersnyder, 2017) and the production of emotional facial behaviors (Bjornsdottir and Rule, 2021). Thus, the purpose of this preliminary study was to examine for the first time whether brain connectivity patterns during perception of emotional faces are a product of two important sources of conceptual knowledge: emotion concept accessibility and one’s cultural background. Specifically, we tested whether emotion words and participants’ cultural background alter functional connectivity between regions implicated in semantic retrieval, visual perception, and social cognition during emotion perception.

There is growing evidence that the availability and accessibility of emotion concept knowledge significantly influences emotion perception (Barrett et al., 2011; Lindquist and Gendron, 2013; Lindquist et al., 2015a; Barrett, 2017, 2022; Nook et al., 2017; Satpute and Lindquist, 2021). An emotion concept such as “disgust” represents a set of variable, situated instances (e.g., disgust when the cat pees on the bed vs. disgust at discovering you have eaten a bite of moldy food vs. disgust about a counter-normative body piercing) that are grounded by modality-specific information tied to the situations in which they occur (e.g., distinctive physiological patterns; Kreibig, 2010; Siegel et al., 2018). According to constructionist models of emotion, emotion concept knowledge is supported via domain-general processes such as abstraction, categorization, and language (Barrett, 2017; Satpute and Lindquist, 2019; Lindquist et al., 2022; see also Xu et al., 2021). Our theoretical framework proposes that emotion category words such as “disgust” serve as placeholders for concept knowledge by cohering together otherwise highly variable situated instances as members of the same abstract category (e.g., Wilson-Mendenhall et al., 2011; Atzil and Gendron, 2017; Doyle and Lindquist, 2018; Hoemann et al., 2019; Hoemann and Barrett, 2019).

Behavioral evidence is consistent with the hypothesis that emotion words support access to emotion concept knowledge, and, in turn, alter the perception of facial muscle configurations (see Lindquist and Gendron, 2013). First, access to emotion words can induce categorical perception for emotional facial behaviors. For example, perceivers who learned to pair chimpanzee facial muscle movements with nonsense words subsequently perceived categorical distinctions between facial behaviors that varied dimensionally in their facial muscle configurations (Fugate et al., 2010). These novel category representations can then shape future perceptions. For example, learning to pair novel facial muscle configurations with a nonsense word caused participants to see a subsequent category exemplar labeled with the same word as more like the learned facial configurations. This effect was significantly reduced when novel facial muscle configurations were initially learned in the absence of nonsense labels (Doyle and Lindquist, 2018). Second, accessibility to emotion words alters the speed and quality of emotion perception. For example, temporarily impeding access to emotion category words leads to slower and less accurate emotion perception when compared to trials on which emotion category words are accessible to perceivers (Gendron et al., 2012; Lindquist et al., 2014). In contrast, priming emotion words leads to faster perceptions that are biased towards category prototypes when compared to trials in which faces are seen without an emotion word (Halberstadt et al., 2009; Nook et al., 2015).

Meta-analyses of human neuroimaging research also show that emotion perception consistently recruits neural regions associated with semantic processing (e.g., Sabatinelli et al., 2011; Lindquist et al., 2012). In particular, in a meta-analysis assessing the impact of emotion words on emotional face processing, Brooks et al. (2017) found that the mere presence of emotion words—such as “anger” or “disgust”—in instructions or as response options in neuroimaging tasks, was associated with greater activity in regions associated with semantic retrieval during subsequent exposure to emotionally evocative stimuli. These findings suggest that the mere presence of emotion category words—as task instructions or response options throughout a task—can serve to prime participants to access emotion concept knowledge that, in turn, influences brain activation during the perception of emotional stimuli (see also Koban et al., 2017). In contrast, during experimental tasks in which emotion words were absent as compared to present, Brooks et al. (2017) found increased BOLD activation in bilateral amygdala extending into the parahippocampal gyrus. These findings are consistent with the hypothesis that words serve as a form of “context” for interpreting emotional faces (e.g., see Lindquist and Gendron, 2013) insofar as amygdala activation has been associated with representing the salience of uncertain stimuli (e.g., Cunningham and Brosch, 2012) and parahippocampal activation has been associated with using context to make meaning of visual objects (Aminoff et al., 2013; Bohbot et al., 2015). More broadly, these findings are consistent with work on “affect labeling,” showing that access to emotion words reduces amygdala activity and the emotional impact of faces during emotion perception (see Torre and Lieberman, 2018; Satpute and Lindquist, 2021 for reviews).

Importantly, emotion categories and the conceptual knowledge they afford differ across cultures (Kitayama et al., 2006b; Gendron et al., 2012; see also Mesquita et al., 2017). Even emotion words considered to be translational equivalents with English emotion categories, such as anger, fear, and happiness, vary significantly in meaning across languages spanning the globe (Jackson et al., 2019). Cross-linguistic variation in emotion category meaning may exist because different cultural groups developed slightly different meanings around common emotionally evocative situations (Gendron et al., 2020; Lindquist et al., 2022; Uchida et al., 2022; see also Uchida et al., 2020; Wang, 2021). Indeed, different cultural groups associate the same emotion category word, such as “disgust,” with different facial configurations (Jack et al., 2016; Fang et al., 2018) and participants from different cultural backgrounds produce different facial muscle movements for the same emotion category (both during spontaneous experience and when explicitly posing those emotion categories; Fang et al., 2022). Furthermore, the neural representation of emotional facial expressions reflects cross-cultural differences in emotion concept knowledge between individuals from Japan versus the United States (Brooks et al., 2019).

More generally, there appear to be culture-based differences in how the brain represents emotional facial behaviors. For instance, past research shows that whereas White participants residing in Japan showed increased activity in the amygdala to White and Japanese faces posing fearful expressions, Japanese participants residing in Japan showed increased activity in the inferior frontal gyrus (Moriguchi et al., 2005). Relatedly, White American participants residing in the United States and Japanese participants residing in Japan showed greater activity in the amygdala to fearful expressions posed by members of their own culture (Chiao et al., 2008). Collectively, these findings suggest that the presence of emotion words and the cultural background of individuals may interact to predict the neural representation of emotion perception. No study to our knowledge, however, has specifically addressed this question. Moreover, past research has examined functional activation magnitude in brain regions, but brain regions do not activate in isolation—brain regions work together as functional neuronal assemblies (e.g., see Pessoa, 2023).

The present study thus tested for the first time whether priming emotion words such as “disgust” alters functional connectivity during emotion perception in Chinese and White American participants residing in the United States. We specifically recruited Chinese individuals who were born and raised in mainland China but now reside in the United States, and non-Hispanic White American individuals who were born and raised in the United States. Participants viewed facial muscle configurations posed by White actors that are stereotypical of the English language emotion categories anger and disgust while undergoing fMRI. Faces portraying behaviors stereotypical of anger and disgust in the United States were used because these emotion categories are both perceived to be associated with unpleasant and highly aroused affective states, and share in the activation of multiple facial muscle groups (see Nook et al., 2015 for a discussion). Emotion concept knowledge may be especially important to the understanding of emotional facial portrayals in such contexts where the portrayals do not differ in valence or arousal (see Widen, 2013; Lindquist et al., 2016; Shablack and Lindquist, 2019 for discussions). Choosing faces with similar muscular activation also allowed us to ensure that brain differences were not merely a product of differences in statistical regularities present in the stimuli.

Across four counterbalanced blocks, posed expressions of anger and disgust were either preceded by the relevant English emotion category word or non-emotional, non-word control text (i.e., “XXXXXX”). English language emotion categories and associated posed facial behaviors were used with the expectation that English emotion words would differentially impact the emotion perception of Chinese participants, who in the absence of priming, might have relatively less automatic accessibility to English language emotion concept knowledge, including the specific facial muscle configurations stereotypically associated with those categories in a United States context (see Fang et al., 2018). Moreover, White actors were used to mimic the majority racial and ethnic identities in the United States broadly, and the local context (the Southeast), specifically. All target actors were self-identified females; we used all female faces since these poses had the highest normed perceiver agreement for the emotion category depicted in the database we used.

Following on the meta-analytic findings of Brooks et al. (2017), we assessed whether the mere presence of emotion category words preceding perception of posed emotional facial behaviors would impact functional connectivity between the left inferior frontal gyrus (IFG) and bilateral amygdala, as well as connectivity of those regions with 70 other regions linked meta-analytically to semantic processing (e.g., Binder and Desai, 2011; Price, 2012), emotion perception (e.g., Lamm et al., 2011; Sabatinelli et al., 2011; Taylor et al., 2012) and social cognition (e.g., Van Overwalle, 2009; Pintos Lobo et al., 2023). Analyses were corrected for multiple comparisons using the false discovery rate.

According to some accounts of emotion perception (e.g., see Ekman and Cordaro, 2011), the mere presence of an emotion category label would have no effect on functional connectivity, nor should it interact with the cultural background of a perceiver to influence perception of so-called universal facial expressions of emotion. On the other hand, constructionist accounts of emotion suggest that a word naming an emotion concept activates a cache of conceptual knowledge about the types of instances that populate that category (e.g., see Barrett, 2006). Even if category words such as “anger” and “disgust” have direct translations in other languages, they are likely associated with different facial muscle movements across people from different cultural backgrounds (e.g., Jack et al., 2016). We thus hypothesized that priming English language emotion categories might differentially impact functional connectivity while Chinese participants raised in China versus White American participants raised in the United States perceived emotional faces. We hypothesized that access to English emotion category words might have a larger impact on functional connectivity in the brains of Chinese participants, since these participants might be less familiar with or have less automatic accessibility to English language emotion concept knowledge. We did not have specific hypotheses regarding the impact of labels on perception of specific emotion categories.

## 2 Methods

### 2.1 Participants

Fifty-one young adults consented to the overall study, but only 45 participated due to time constraints. All participants were right-handed and denied any history of neurological or psychiatric disease. Participants consented to the study as approved by the UNC Institutional Review Board and were compensated $50 for their involvement. Four participants were excluded due to head motion exceeding 2 mm. Four additional participants were unable to complete the scanning session in the time allotted due to experimental errors. One other participant requested to leave the scanner mid-scan due to claustrophobia. Thus the final count of participants in this study was 36, comprising 15 Chinese participants (*M*_age_ = 20.4 ± 2.2; 9 self-identified females) and 21 White American participants (*M*_age_ = 22.3 ± 3.4; 10 self-identified females). No age differences were observed between self-identified female and male participants (*b* = −1.54, *SE* = 0.96, *t* = −1.61, *p* = 0.118), cultural groups (*b* = −1.71, *SE* = 0.96, *t* = −1.78, *p* = 0.085), and self-identified male and female participants within cultural groups [*F*_(1,32)_ = 2.69, *p* = 0.111].

White American participants were born and raised in the United States by primarily monolingual English-speaking non-Hispanic White American-born parents, and all denied ever residing outside of the United States. In contrast, Chinese participants were born and raised in provinces of mainland China excluding areas with significant Western influence, such as Hong Kong, Macau, and Taiwan. These participants were raised by primarily monolingual Mandarin-speaking Chinese-born parents, and all denied ever residing outside their provinces prior to arriving in the United States as adults. In addition, given that Chinese participants had resided in the United States for an average of less than 20 months (*M*_months_ = 18.4 ± 15.1), they were required to undergo the Test of English as a Foreign Language (TOEFL; Educational Testing Service) to assess their proficiency in English communication. All Chinese participants demonstrated the highest level of proficiency in reading, listening, speaking, and writing in English (*M*_TOEFL_ = 107.6; range = 102–112). No significant differences were observed between self-identified Chinese female and male participants in terms of their duration of stay in the United States [*F*_(1,13)_ = 0.02, *p* = 0.879] or TOEFL scores [*F*_(1,13)_ = 0.34, *p* = 0.572].

### 2.2 Practice task

All participants underwent two practice runs of the fMRI task outside the scanner on a laptop computer. These practice runs were identical to the actual fMRI task with the exception of the emotion labels and posed emotional facial portrayals that were used; we opted to use the category “sadness” so as not to impact participants’ perceptual representation of posed angry and disgusted faces prior to seeing them in the scanner. The first practice corresponded to the emotion-word label condition and participants saw the word “Sadness,” after which they passively viewed images of actors portraying facial muscle configurations stereotypical of sadness in the United States. The second practice run corresponded to the control condition. This practice run was exactly like the first but the emotion label was replaced with a control text with no semantic meaning: “XXXXXX.”

### 2.3 Study design

**Figure 1:**
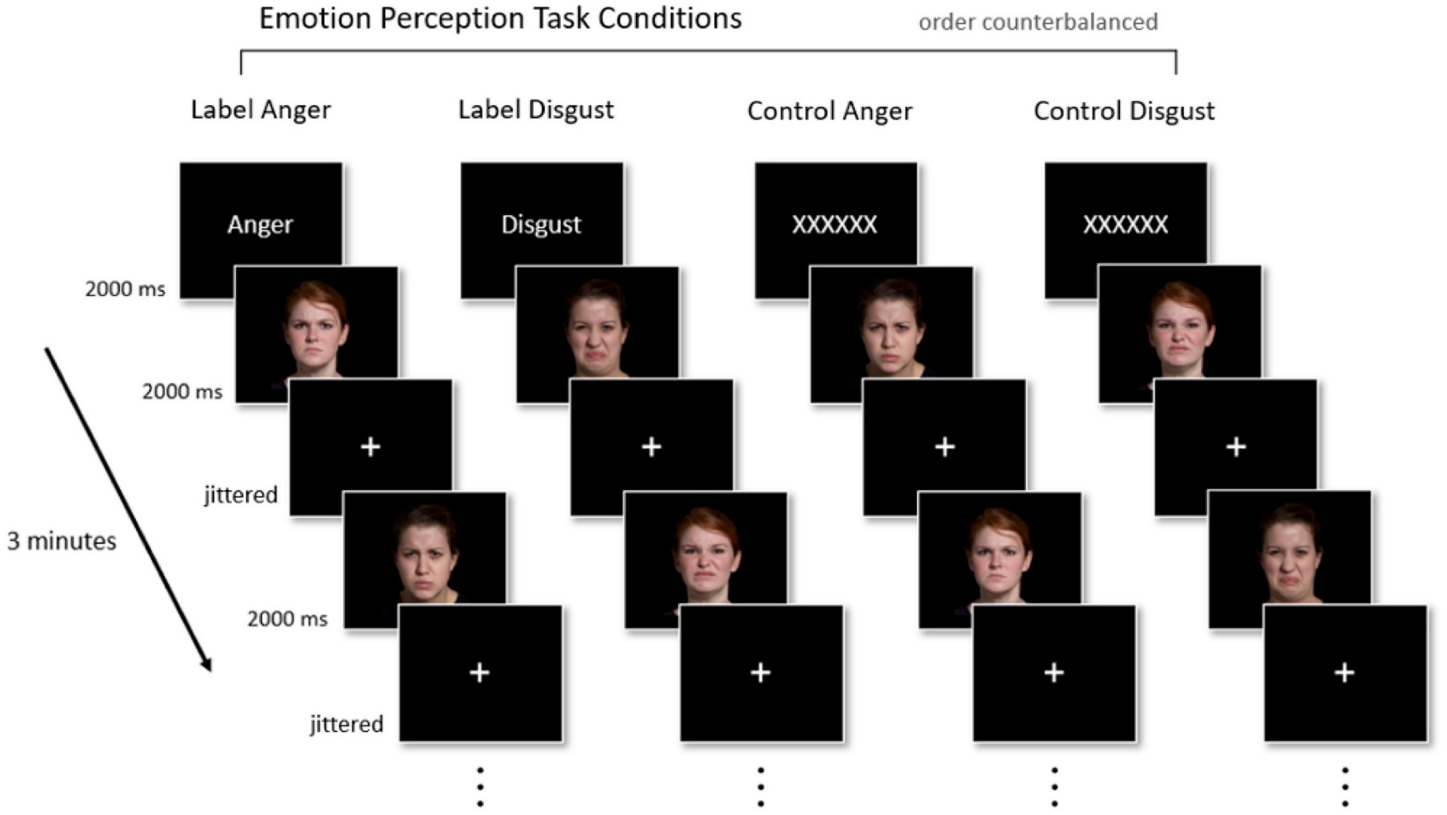
Experimental paradigm. Participants completed four 3-min fMRI runs in a 2 × 2 block design (Face Expression [Anger, Disgust] × Prime Condition [Emotion Label, Control Text]). Priming (i.e., the presentation of either an emotion concept or a string of Xs) occurred once for 2000 ms at the start of every run. Participants then passively viewed 40 faces, each shown for 2000 ms, displaying either anger or disgust; faces corresponding to anger or disgust only appeared in their respective labeled run or control run. Jittered fixation crosses (2000–8000 ms) were used as interstimulus intervals. Facial images reproduced with permission from IASLab Face set (www.affective-science.org). The IASLab Face Set Release Agreement permits the display of only two specific actors when showcasing examples from the stimulus set in publications (i.e., the actors shown in this figure). In the present study, however, we used stimuli from 10 actors.

Participants underwent four 3-min fMRI runs in a 2 (Face Expression: Anger vs. Disgust) x 2 (Prime Condition: Emotion Label vs. Control Text) block design. In two of the four runs, participants saw one emotion-word label (“Anger” in one run, “Disgust” in the other) prior to the start of the run. In the other two runs, the emotion word was replaced with a Control Text (“XXXXXX”). In both the Emotion Label and Control Text conditions, text was only shown once at the start of each fMRI run for 2000 ms in order for the priming effect to remain subtle. Following the Emotion Label or Control Text, participants passively viewed images of actors portraying facial muscle configurations stereotypical of anger or disgust in the United States. We assessed passive viewing because we were interested in whether the mere presence of the emotion word label impacted activation in regions involved in semantic retrieval, even when participants were not explicitly asked to render a category judgment about the face at any point in time during the task. In each block, 40 faces were presented per block for 2000 ms each. Fixation crosses served as interstimulus intervals (ISIs) and remained on-screen for a jittered amount of time (2000–8000 ms). Because we were interested only in conditions in which an emotion word was congruent with the pictured facial muscle configurations, facial configurations stereotypical of anger were only shown in the “Anger” run [Label Anger] and in one “XXXXXX” run [Control Anger]. Similarly, facial configurations stereotypical of disgust were only shown in the “Disgust” run [Label Disgust] and one “XXXXXX” run [Control Disgust]. The four runs were counterbalanced and faces were presented in a random order within each block. Figure 1 illustrates the fMRI paradigm.

### 2.4 Stimuli

Face stimuli were taken from the NimStim Face set (Tottenham et al., 2009) and the IASLab Face set.^1^ We selected 10 different faces expressing sadness from the NimStim Face set to be shown during the practice task. Face stimuli shown in the fMRI emotion perception task were collected from separate data sets in order to control for potential priming effects prior to scanning. For the fMRI emotion perception task, we used the IASLab Face set to select 10 images of actors portraying facial muscle configurations stereotypical of anger and 10 images of the same actor portraying facial muscle configurations stereotypical of disgust. As a result, the two emotion conditions presented repeated instances of the same actor from the IASLab Face set. All images depicted White women expressing emotions with a closed mouth to reduce the additional impact of race and gender on emotional face representations and, more practically, because White women’s posed facial behaviors had the highest normed inter-rater agreement for the intended emotion category in the database we used.

### 2.5 fMRI data acquisition

We used a 3 Tesla Siemens PRISMA whole-body scanner to acquire structural images and fMRI data. The first structural image was a T1*magnetization-prepared rapid-acquisition gradient echo: slice thickness = 0.8 mm; 208 slices; repetition time (TR) = 2400 ms; echo time (TE) = 2.22 ms; matrix = 320 × 320; field of view (FOV) = 256 mm; voxel size = 0.8 × 0.8 × 0.8 mm^3^; sagittal plane. The second structural image was a T2*-weighted, matched-bandwidth, high resolution, anatomical scan: slice thickness = 3 mm; 38 slices; TR = 5700 ms; TE = 65 ms; matrix = 192 × 192; FOV = 230 mm; voxel size = 1.2 × 1.2 × 3.0 mm^3^. The functional images were T2*-weighted echo-planar images: 37 slices; slice thickness = 3 mm; TR = 2000 ms; TE = 25 ms; matrix = 92 × 92; FOV = 230 mm; voxel size = 2.5 × 2.5 × 3.0 mm^3^.

### 2.6 fMRI data preprocessing

fMRI data were preprocessed using SPM12 (Welcome Trust Centre for Neuroimaging at UCL, London, UK), implemented in MATLAB 2018a (Mathworks Inc., Natick, MA). Volumes were slice-time corrected, realigned to the mean volume to correct for head motion, normalized, and warped into the standard stereotactic space defined by the Montreal Neurological Institute (MNI, 2 mm). We processed image artifacts originating from head movement using the ART-based scrubbing procedure as an artifact removal tool (Nieto-Castanon, 2020). Signal contributions from the white matter, cerebrospinal fluid, linear BOLD signal trends within each session, and micro-head movements (12 parameter estimates: 3 translation, 3 rotation, and their associated first-order derivatives) were regressed out of the data. Lastly, the fMRI data were band-pass filtered (0.008–0.09 Hz) and functional images were spatially smoothed using a Gaussian filter kernel (full width at half-maximum = 8 mm) for subsequent ROI-to-ROI analyses.

### 2.7 Generalized psychophysiological interaction analysis

Functional connectivity was analyzed with the CONN toolbox (version 18b; Whitfield-Gabrieli and Nieto-Castanon, 2012) in MATLAB R2018a (Mathworks Inc., Natick, MA) using generalized psychophysiological interaction (gPPI). The seeds of interest were bilateral amygdala and left IFG; meta-analytically, the left IFG shows consistent activation across fMRI studies on emotion perception when emotion concepts, relative to control concepts (i.e., gender concepts), are present in the fMRI task as instructions or response options (Brooks et al., 2017). These findings were taken as evidence by Brooks et al. (2017) that emotion perception requires relatively greater access to semantic knowledge than gender perception. In contrast, bilateral amygdala shows consistent activation for the inverse contrast across fMRI studies on emotion perception (Brooks et al., 2017). These findings were taken as evidence by Brooks et al. (2017) that in the absence of emotion category words, emotional facial expressions are more ambiguous in meaning.

We used the Schaefer atlas to identify a parcellation for the left IFG seed using peak coordinates from Brooks et al. (2017). We chose the Schaefer atlas for its ability to provide homogeneous and neurobiologically meaningful features of brain organization based on a multiresolution parcellation generated from using both task-fMRI and resting-state fMRI data across diverse acquisition protocols (Schaefer et al., 2018). Because the Schaefer atlas lacks subcortical parcellations, bilateral amygdala seeds were constructed using peak coordinates of amygdala activation from our meta-analysis on the brain basis of emotion (see Lindquist et al., 2012; Supplementary Table S3). ROIs were constructed as 6 mm spheres using the MarsBarR toolbox for SPM (Brett et al., 2011) centered at the peak coordinates.

Target regions were selected via the CONN toolbox, which uses both the Harvard-Oxford atlas and AAL atlas (Tzourio-Mazoyer et al., 2002) for cortical and cerebellar parcellations. We specifically were interested in regions that, meta-analytically, show consistent activation during semantic retrieval (e.g., Binder and Desai, 2011; Price, 2012), social cognition (e.g., Van Overwalle, 2009; Pintos Lobo et al., 2023), and emotion perception (e.g., Lamm et al., 2011; Sabatinelli et al., 2011; Taylor et al., 2012). Target regions, in no particular order, spanned superior frontal gyrus (bilateral), middle frontal gyrus (bilateral), right inferior frontal gyrus (pars triangularis and opercularis), temporal poles, superior temporal gyrus (bilateral), middle temporal gyrus (bilateral), superior parietal lobule (bilateral), supramarginal gyrus (bilateral), angular gyrus (bilateral), medial prefrontal cortex, anterior cingulate gyrus (bilateral), anterior insula (bilateral), precuneus (bilateral), parahippocampal gyrus (bilateral), lingual gyrus (bilateral), fusiform gyrus (bilateral), and the cerebellum (crux and vermis). Many of these regions are additionally activated during studies of emotion in general (Kober et al., 2008; Lindquist et al., 2012) and emotion perception, in particular (Fusar-Poli et al., 2009).

First-level ROI-to-ROI gPPI analysis was then implemented in CONN to examine how emotion labels (anger, disgust) and control text (XXXXXX) modulate functional connectivity during emotion perception between seed and target regions. A gPPI analysis computes how functional association strength between a seed region (e.g., IFG) and a target region (e.g., precuneus) covaries with an external or experimental factor, such as task conditions. In CONN, gPPI analysis involves computation of separate multiple regression models for each target region BOLD timeseries; this involves (a) all of the selected task effects convolved with a canonical hemodynamic response function (psychological term), (b) seed ROI BOLD timeseries (physiological term), and (c) the interaction term specified as the product of (a) and (b) (PPI term). Second-level analyses were then performed to control for multiple comparisons at the level of seeds using parametric statistics based on Gaussian Random Field Theory (Worsley et al., 1998). Cultural group was used as a covariate in the second-level analysis, with a contrast of *Chinese > White American* set for each of the seed regions for differences between task conditions, that is, *Anger Label > Anger Control* and *Disgust Label > Disgust Control*. We used the false discovery rate (FDR) method for correction for multiple comparisons (*p* < 0.05, two-tailed) (Genovese et al., 2002).

## 3 Results

We found no significant difference in functional connectivity between seed regions (bilateral amygdala and left IFG) and target regions during the anger label condition relative to the anger control text condition. Moreover, there were no differences between cultural groups in functional connectivity for the anger conditions.

**Figure 2:**
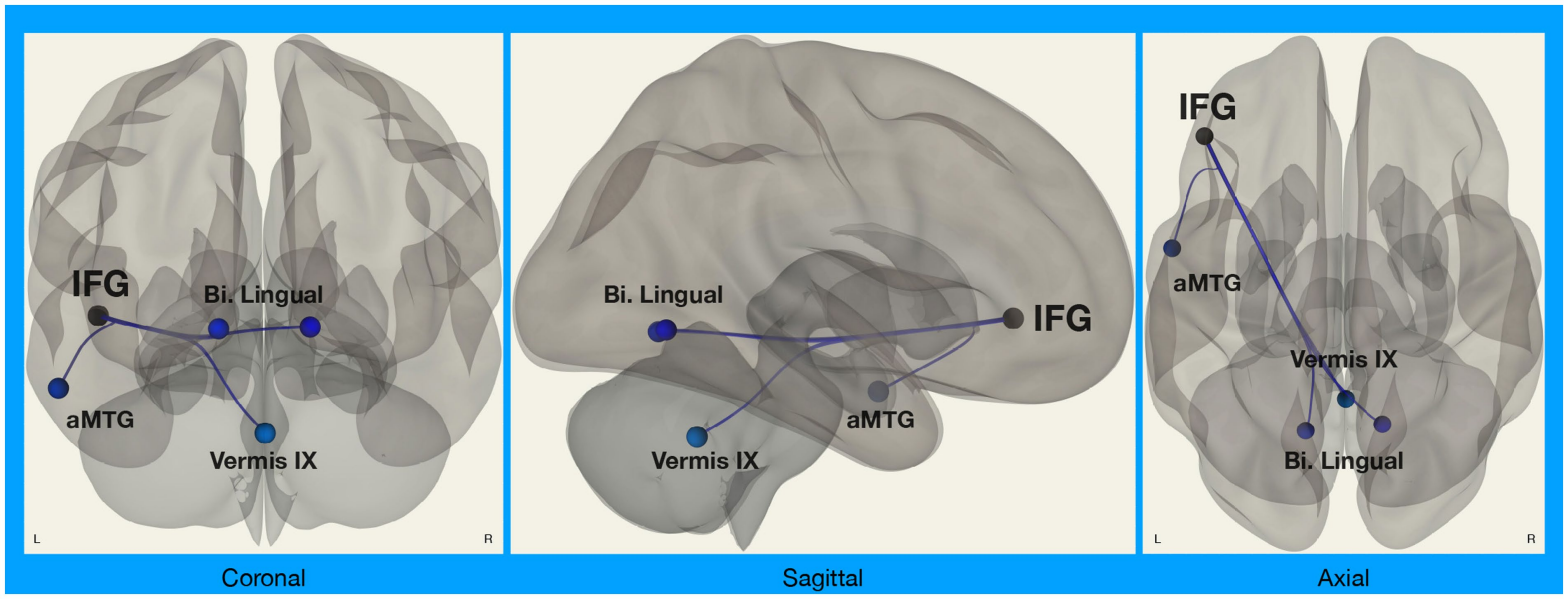
Connectivity between left IFG and target regions. Functional connectivity analyses involved seed regions (3) and target regions (70); these analyses were corrected for multiple comparisons using the false discovery rate (seed-level FDR-corrected *p* < 0.05). Results showed significant differences in functional connectivity between the left inferior frontal gyrus (IFG; a seed region) and several target regions (Bi. Lingual, bilateral lingual gyri; aMTG, left anterior middle temporal gyrus; Vermis IX, cerebellar vermis 9) during disgust label vs. control text in Chinese participants compared to White American participants.

**Figure 3:**
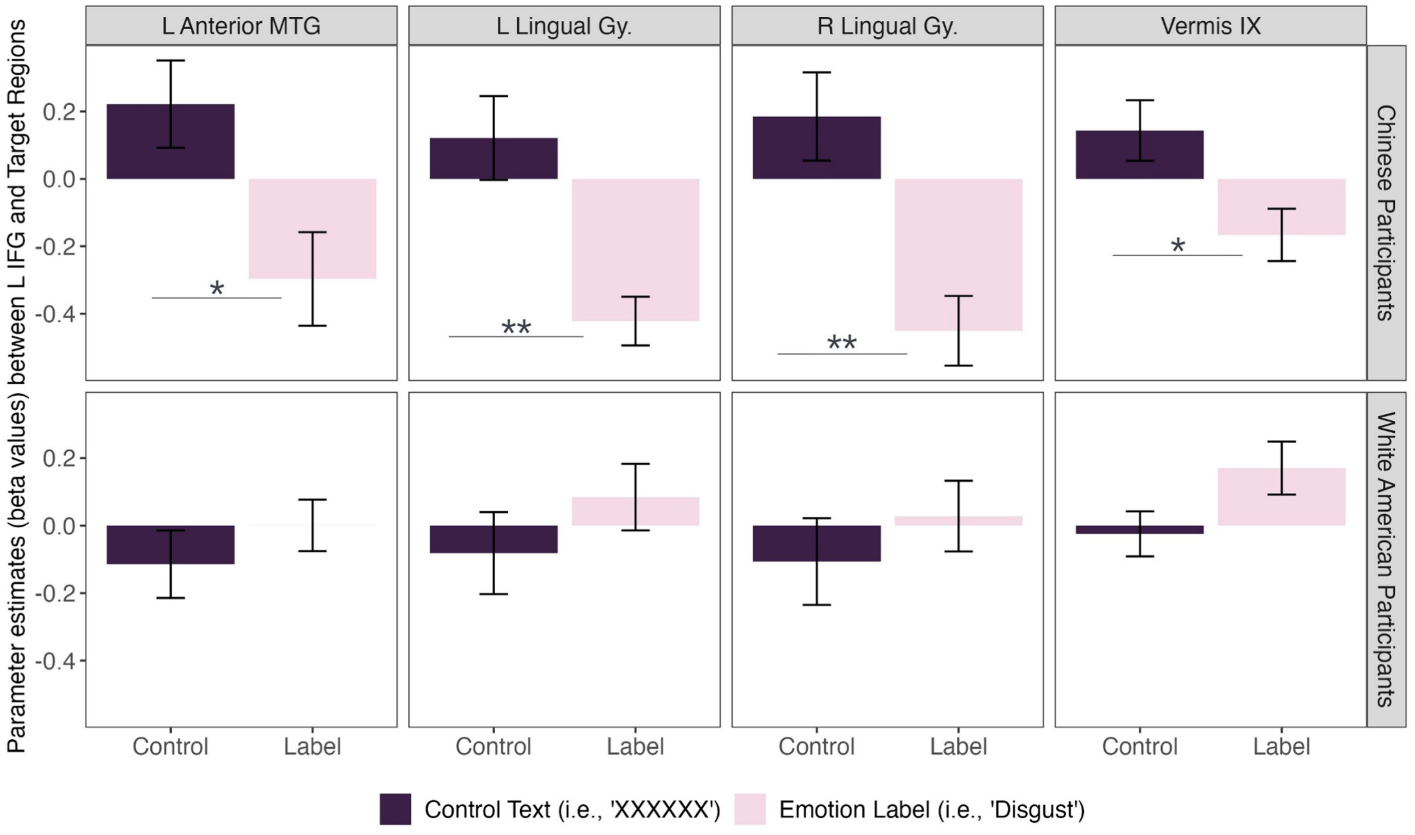
Connectivity differences between cultural groups during disgust perception. Chinese participants **(top row)** showed significant functional connectivity between the left inferior frontal gyrus (L IFG) and left anterior middle temporal gyrus [*t*(34) = −3.53], bilateral lingual gyri [left: *t*(34) = −4.23; right: *t*(34) = −4.81], and cerebellum [i.e., vermis IX; *t*(34) = −3.33] during disgust perception when primed with an emotion label (i.e., “disgust”; light pink) compared to control text (i.e., “XXXXXX”; dark purple). In contrast, there was insufficient evidence to conclude that White American participants **(bottom row)** showed a significant difference in functional connectivity between prime conditions for disgust perception. Asterisks: * = *p* < 0.05, ** = *p* < 0.01.

We did, however, observe significant differences in functional connectivity between left IFG and target regions in the *Disgust Label* > *Control Text* for Chinese compared to White American participants (Figure 2). Specifically, we found that functional connectivity between the left IFG and regions implicated in visual face perception (bilateral lingual gyrus), mentalizing (vermis IX), and semantic representation (middle temporal gyrus) decreased in the emotion label condition relative to the control text condition for Chinese participants only [*F*_(8,27)_ = 2.58, *p* = 0.031; *p* < 0.05, two-sided FDR seed-level correction] (Figure 3).

We found no significant difference in functional connectivity between bilateral amygdala and any target regions between conditions or cultural backgrounds.

## 4 Discussion

How culture plays a role in the neural representation of emotion perception—and whether language interacts with culture in this process—is a question of enduring interest in affective neuroscience. Yet very little research has explicitly examined this topic. This preliminary study of 36 participants from the United States and mainland China is one of the first to explicitly examine how access to emotion concept knowledge interacts with a person’s culture of origin to impact the neural representation of emotional faces. We based our study on Brooks et al. (2017) meta-analysis examining the effect of emotion word priming on the neural representation of emotion perception. The studies represented in Brooks et al. (2017) did not explicitly manipulate the presence or absence of emotion category words in experimental tasks, but, when meta-analyzed, showed that emotion category word accessibility nonetheless influenced the neural representation of emotional stimuli. To follow up on this work, we explicitly primed participants from different cultural backgrounds with English language emotion category word labels (or non-word controls) before emotion perception. We predicted that priming emotion words might especially influence functional connectivity for participants of Chinese descent living in the United States because labels would help them access emotion concept knowledge consistent with their English-speaking host culture. We focused on seed regions of interest observed in Brooks et al. (2017): the left IFG and bilateral amygdala. We found that culture exerted an effect on the functional connectivity between IFG and regions implicated in visual perception, semantic representation, and social cognition for Chinese participants only, and only when the word “disgust” was primed prior to perceiving White actors’ faces portraying disgust. This finding suggests that both culture and access to emotion category words may influence how the brain represents emotional facial behaviors during emotion perception. These findings converge with other growing behavioral (Barrett, 2006; Gendron et al., 2012; Lindquist and Gendron, 2013; Nook et al., 2015; Lindquist et al., 2015a,b; Satpute and Lindquist, 2021) and neural (Brooks et al., 2017, 2019; Brooks and Freeman, 2018) evidence that conceptual knowledge in the mind of perceivers plays an important role in emotion perception. They also add to a relatively small cultural neuroscience literature examining cross-cultural differences in emotion perception.

### 4.1 Cultural influences on emotion perception

The effects of culture on emotion perception found in the present study help to inform the current literature on cultural neuroscience (see Han and Ma, 2014; Shkurko, 2020). Our finding that priming the word “disgust” influenced functional connectivity for Chinese participants, but not White American participants, suggests that access to emotion words had a differential effect for people from different cultural backgrounds. It may be that White American participants’ functional connectivity during emotion perception did not differ as a product of whether a word did or did not precede the perception of posed facial emotional behaviors because emotion concept knowledge associated with English emotion words is more chronically accessible for White Americans who speak English than Chinese from mainland China who are recent immigrants to the United States.

Although we made no predictions about whether specific emotion categories would show differential functional connectivity between the experimental conditions under study, our results are interesting in light of evidence that disgust is expressed (Fang et al., 2022) and perceived (Fang et al., 2018) as less distinctive than anger in Chinese versus White European participants. Moreover, translations of the English category “disgust” do not exist in traditional Daoist, Buddhist, or Confucian Chinese texts (Russell and Yik, 1996), suggesting that the category might have been traditionally less central to Chinese culture than to cultural groups descending from Western Europe.

Our findings suggest that in the absence of explicit access to the English emotion word “disgust,” Chinese participants were processing facial behaviors associated with the category disgust differently than when they had access to the word. Past research associates lingual gyrus activation with face perception (Watson et al., 2016), middle temporal gyrus activation with categorization and semantics (Buckley et al., 1997; Visser et al., 2012), and the vermis 9 of the cerebellum with mentalizing (Van Overwalle et al., 2020). Thus, although speculative, these findings may suggest that providing Chinese participants with the English label “disgust” reduced their need to engage in elaborative meaning making of the disgusted facial behaviors posed by White American actors by drawing on visual information processing, semantic retrieval, and social cognition. Additionally, priming access to the relevant English category may have allowed Chinese participants to easily access the English concept of “disgust” to resolve the meaning of White Americans’ facial behaviors. It is possible that we did not find this effect for posed angry faces because the facial behaviors associated with anger in the United States are either more like those associated with anger in China, or because Chinese participants living in the United States are merely more familiar with facial behaviors associated with anger in the United States. Future research should thus examine how familiarity with the facial behaviors prototypically associated with certain emotion categories in the host culture and a participant’s degree of acculturation impact these findings.

### 4.2 Limitations and future directions

To our knowledge, this is the first study to test hypotheses about the impact of language on functional connectivity during emotion perception. Our findings should thus be viewed as preliminary evidence and a concept proof that language and culture may together influence the neural representation of emotion perception. The current study was limited in multiple ways that should be improved upon in future research. First, there are limitations of our design that should be noted. Priming conditions were explicit, albeit subtle and fleeting; participants were not given expectations for the relevance of the words, and they only viewed them for 2 s before seeing a number of same-category faces. This allowed us to test whether mere exposure to category information changed subsequent processing of faces, even when there was no goal to explicitly categorize those faces.

We chose non-words (XXXXX) as our control condition rather than using control words with semantic meaning to most closely mimic Brooks et al. (2017)‘s meta-analysis in which the presence of emotion words was compared to the absence of emotion words. Including controls with semantic meaning also could have biased perception in unknown ways. Fortunately, the fact that we found effects specific to disgust in Chinese participants suggests that our findings are not just due to the effect of viewing any word versus non-words.

We also assessed passive viewing as opposed to including an active task because we were interested in whether the mere presence of the emotion word label impacted activation in regions involved in semantic retrieval, visual perception, and social cognition, even when participants were not explicitly asked to render a category judgment about the face. This meant that we could not ensure that participants were actively categorizing the faces as emotional, but it also rules out that our findings are merely due to task demands for explicit categorization. Our design was thus, in many ways, a subtle and conservative test of our hypotheses. The fact that there was an effect of any of the labels—especially on Chinese participants’ brain connectivity—during perception of disgust is suggestive that the prime was sufficient to alter subsequent processing of the faces. Again, the fact that we found connectivity differences between the label and control condition when viewing disgust faces suggests that participants were likely paying attention to these faces, but future research should replicate these findings with a range of passive and active conditions.

Second, there are limitations associated with our sample. While our sample size aligns with those of many cultural neuroscience studies (e.g., Freeman et al., 2009; Adams et al., 2010; Cheon et al., 2011; de Greck et al., 2012; Immordino-Yang et al., 2014; Park et al., 2017; Qu and Telzer, 2017), it is modest compared to broader neuroimaging standards. Consequently, this may have reduced our ability to detect subtle effects, especially at the whole-brain level given the strict statistical thresholds inherent to neuroimaging (see Chen et al., 2020, for a discussion). The absence of significant effects in our functional activation results further underscores this limitation (see Supplementary materials). Nevertheless, it is important to note that these null findings—from both functional activation and connectivity results—should not be interpreted as definitive evidence against certain effects. A larger sample may yield different insights. As noted earlier, many cultural neuroscience studies with similar sample sizes have been replicated and validated through systematic literature reviews (e.g., see Han and Ma, 2014; Shkurko, 2020). Central to our study are the significant effects highlighting the role of culture and concept accessibility on functional connectivity during emotion perception, whose corresponding hypotheses are grounded in meta-analyses of the affective neuroscience literature (e.g., Sabatinelli et al., 2011; Lindquist et al., 2012; Brooks et al., 2017). Our findings provide preliminary evidence supporting the notion that the neural underpinnings of emotion perception are contingent on the mind of the perceiver. Future research, employing larger samples, will need to investigate and assess the consistency of these effects.

Moreover, we selected our sample to be prototypical of the East–West paradigm commonly used in cross-cultural psychology studies of emotion (e.g., see Mesquita et al., 2017). Yet there are limitations associated with these two-culture comparisons. Future studies interested in similar effects of emotion-word labels and culture may find it informative to utilize continuous and multiple discrete measures of culture. We also sampled individuals of Chinese descent who were living in the United States, which meant they were not completely naïve to White American facial emotional expressions. These individuals might also be different from Chinese individuals who have not moved to the United States on a number of dimensions including personality (Kitayama et al., 2006a) or levels of acculturation to US emotional norms (see Zhou et al., 2021). By selecting participants from a wider pool of Chinese with greater variation in time spent in the US, future research could also specifically examine the effects of acculturation. There is evidence that emotion concept understanding (Jackson et al., 2019), facial expressions (Niedenthal et al., 2019), and patterning of emotional experiences (De Leersnyder, 2017), may evolve as a product of cross-cultural contact. Potential future studies may also benefit from incorporating additional conditions such that there are same-race stimuli present for each cultural group and there are labels used in each participant’s primary language. Such a paradigm could reveal inter-group biases as well as an additional benefit of labels from participants’ primary versus secondary language (e.g., see El-Dakhs and Altarriba, 2018).

## 5 Conclusion

Our findings add to growing evidence that conceptual knowledge activated in the minds of perceivers influences emotion perception. We provide preliminary evidence that brain representations of emotional facial expressions are influenced by two important sources of conceptual knowledge: a person’s access to emotion category words and their cultural background. We assessed the neural processes involved in emotion perception in a sample of Chinese and White American participants living in the United States. Our findings that functional connectivity associated with emotion perception differs as a product of cultural background and access to the host culture’s emotion concepts are especially relevant in a rapidly globalizing society in which individuals from different cultural groups live in the same context.

## Data availability statement

The datasets presented in this study can be found in online repositories. The names of the repository/repositories and accession number(s) can be found at: https://osf.io/7wfej/?view_only=e2aa8a5c2a6f4d74a7355b31d8019156.

## Ethics statement

The studies involving humans were approved by The University of North Carolina at Chapel Hill’s Institutional Review Board. The studies were conducted in accordance with the local legislation and institutional requirements. The participants provided their written informed consent to participate in this study.

## Author contributions

JL: conceptualization, methodology, investigation, supervision, data curation, software, formal analysis, visualization, writing-original draft preparation, and writing-review and editing. MC: conceptualization, methodology, data curation, software, formal analysis, visualization, writing-original draft preparation, and writing-review and editing. CD: conceptualization, methodology, investigation, supervision, data curation, software, and formal analysis. KL: conceptualization, funding acquisition, methodology, resources, writing-original draft preparation, writing-review and editing, supervision, and project administration. All authors contributed to the article and approved the submitted version.

## Footnotes

^1^www.affective-science.org
