# Supplementary material for: Language access differentially alters functional connectivity during emotion perception across cultures
Source: Front Psychol. 2024 Feb 15;14:1084059. doi: 10.3389/fpsyg.2023.1084059 (PMC10901990; doi:10.3389/fpsyg.2023.1084059)
Supplement: Supplementary file 1 [file Data_Sheet_1.PDF]

Supplementary Material

Whole-brain and Regional Brain Activation Results

1 Background

Emotion concept knowledge in the form of emotion words such as “anger” or “disgust” influences emotion perception (Barrett, 2006; Lindquist et al., 2015; Lindquist & Gendron, 2013; Satpute & Lindquist, 2021). During fMRI studies on emotion perception, the presence of emotion words in the experimental context (e.g., in a forced-choice task) activates regions associated with semantic processing, while the absence of these words activates regions associated with uncertainty (Brooks et al., 2017). These brain activation patterns, consistent across various emotion perception tasks (Brooks et al., 2017), suggest a neurobiological mechanism whereby retrieval of semantic information (i.e., language to make meaning of a facial expression) reduces the ambiguity of the sensory input by refining it as a specific emotion category (e.g., “an angry face”) (see Satpute & Lindquist, 2019, 2021, for discussions; see also Betz et al., 2019).

To our knowledge, Brooks et al. (2017)’s meta-analytic findings that the presence of emotion words in the experimental context influences neural responses associated with emotion perception have yet to be tested experimentally. Hence, we sought to replicate and extend Brooks et al.’s findings by testing the hypothesis that the neural basis of emotion perception depends in part on the accessibility of emotion concept knowledge.

Thus we manipulated participants’ ($N = 36$) accessibility to emotion concept knowledge by priming participants with either emotion words (“anger”, “disgust”) or control text (“XXXXXX”) prior to viewing facial configurations of prototypical North American expressions of anger and disgust. We also investigated the effect of participants’ cultural background (Chinese v. White American) on the neural basis of emotion perception, given that a person’s cultural upbringing significantly shapes the development of their emotion concept knowledge (Chiao, 2018; Gendron et al., 2020; Lindquist et al., 2022). We tested our hypothesis using both functional activation and connectivity methods; this supplement focuses on the activation results.

Please refer to the main text for details on the sampled participants and experimental design.

2 Hypotheses and Analyses

This supplement examines whether the presence of emotion words (“anger”, “disgust”) influences neural responses (functional activation) associated with emotion perception and whether these effects vary between cultural groups (Chinese v. White American). We hypothesized: (a) that English emotion words might affect Chinese participants differently due to potentially lesser accessibility to English emotion concepts, including associated facial configurations; (b) that emotion-word priming would lead to increased activation in regions linked to semantic retrieval and processing (e.g., left inferior frontal gyrus) compared to control-text priming; and (c) that control-text priming would lead to increased activation in regions linked to uncertainty (e.g., bilateral amygdala) compared to emotion-word priming.

We used a univariate whole-brain activation and a region-of-interest (ROI) approach to investigate these hypotheses. ROIs and hypothesized effects are outlined in Supplementary Tables 1 and 2. We held no specific predictions regarding how labels would affect perceptions of different emotions across cultures.

Note that in the main text we focus on similar hypotheses but through a functional connectivity lens, highlighting large-scale neural connections over isolated activation points. These methodological divergences can yield different results.

Please refer to the main text for details on fMRI data acquisition and pre-processing.

2.1 Whole-brain Analysis

After data pre-processing, individual subject statistical maps were generated using a general linear model (GLM), in which onsets and durations were defined based on the affective stimuli (face) presentations. Two variables—Emotion Category (Anger, Disgust) and Prime Type (Emotion Label, Control Text)—were explicitly modeled in the design matrix. The baseline condition consisted of the jittered interval trials and was implicitly modeled.

A GLM subject-level model was created for each condition—[Label Anger], [Label Disgust], [Control Anger], and [Control Disgust]—and for contrasts that examined neural responses associated with: the impact of emotion labels on the perception of anger, [Label Anger v. Control Anger]; the impact of emotion labels on the perception of disgust, [Label Disgust v. Control Disgust]; and the impact of emotion labels on the perception of anger or disgust, [(Label Anger + Label Disgust) v. (Control Anger + Control Disgust)]. These GLMs were subsequently included in a second-level mixed-effects model, treating subjects as the random effect. Whole-brain results applied a threshold of $p < .001$ (voxel-wise) and underwent FDR correction at $p < .05$.

2.2 ROI Analysis

Regional brain activation was investigated using ROIs created in FSLeyes (version 1.0.13). ROIs were 6 mm spheres centered at the MNI coordinates in Supplementary Table 1. Mean parameter estimates were extracted from these ROIs for each condition: [Label Anger], [Label Disgust], [Control Anger], and [Control Disgust]. Subsequent reformatting transformed the parameter estimates from wide-form to long-form to structurally represent the repeated-measure design of the fMRI experiment. This latter step created a new factor—ROI—and a single outcome variable: regional brain activation.

We then fit a single mixed-effects model. Regional brain activation was regressed on emotion category, prime type, culture, ROI, and the interactions between these factors. Age and self-identified (biological) sex were added as covariates. Within- and between-subject factors were treated as fixed effects; individual subjects were treated as random effects. ANOVA was subsequently used to examine the sequential decomposition of the contributions of the fixed-effects terms (Bates et al., 2015).

Statistical analyses were carried out using R 4.2.2 (R Core Team, 2022) and *tidyr* (v1.2.0; Wickham & Girlich, 2022), *dplyr* (v1.0.9; Wickham et al., 2022), *lmerTest* (v.3.1-3; Kuznetsova, 2017), and *emmeans* (v1.7.4-1; Lenth, 2022) packages. The full reproducible code is available at OSF: https://osf.io/7wfej/?view_only=e2aa8a5c2a6f4d74a7355b31d8019156.

3 Results

3.1 Whole-brain Results

Modeled conditions—[Label Anger], [Label Disgust], [Control Anger], [Control Disgust]—showed significant activation at the whole-brain level (relative to baseline) in regions associated with face perception, including bilateral fusiform gyrus and hippocampus (Supplementary Figure 1). No significant activation emerged for the modeled contrasts.

3.2 ROI Results

Neither emotion category (anger, disgust) nor prime type (emotion label, control text) showed a significant effect on regional brain activation during emotion perception. These factors also showed no significant interaction with culture (Chinese v. White) nor ROI. Culture and ROI, however, showed significant variation in regional brain activation during emotion perception. White American participants showed significantly greater regional brain activation throughout the task than did Chinese participants ($b = 0.17$, $SE = 0.06$, $t_{(32)} = 2.99$, $p = .005$), but participants overall showed significantly greater activation throughout the task in the left inferior frontal gyrus and bilateral amygdala than activation in other regions (Supplementary Figure 2).

4 Discussion

We found no support for our hypothesis that concept knowledge in the form of emotion words influences emotion perception at the level of functional brain activation. These null effects are likely due to power. Brooks et al. (2017) relied on a meta-analysis approach, aggregating results across multiple studies and thus benefitting from increased statistical power. Our sample's size might partially explain the divergence in findings, as our study might not have had sufficient power to detect functional activation patterns as observed in Brooks et al. (2017).

We did, however, find that participants showed significantly greater activation throughout the task in two particular regions relative to all other regions: the inferior frontal gyrus and amygdala. While purely speculative, this might indicate that participants were both interpreting the affective facial stimuli and reacting to the inherent saliency of faces as stimuli. In some cases, especially during the control priming, the facial stimuli could have presented ambiguous emotional cues. These significantly activated regions—the left inferior frontal gyrus and bilateral amygdala—and their connectivity to the rest of the brain during emotion perception are the focus of the main text.

5 Supplementary Tables

ROIs and Hypothesized Effects for Whole-brain and Regional Brain Activation

Hypothesized Effects	H	ROI	x	y	z
Label > Control	R	Superior Temporal Gyrus	48	-18	1
	R	Middle Temporal Gyrus	57	-22	-9
	L	Inferior Frontal Gyrus	-32	6	-9
Control > Label	R	Amygdala	25	-9	-10
	L	Amygdala / Parahippocampal Gyrus	-25	-1	-16
	L	Parahippocampal Gyrus	-14	-13	-13

Supplementary Table 1. Hypotheses are based on Brooks et al. (2017). ROIs were sourced from Table 2 in Brooks et al. (2017), with x, y, and z representing the MNI coordinates used for their construction. H = Left (L), Right (R) cerebral hemisphere. Label = Emotion Label, Control = Control Text.

Hypotheses for Regional Brain Activation Related to Culture

Hypothesized Effects	H	ROI
(Label > Control)$_{CA}$ >	R	Superior Temporal Gyrus
(Label > Control)$_{WA}$	R	Middle Temporal Gyrus
	L	Inferior Frontal Gyrus
(Control > Label)$_{CA}$ >	R	Amygdala
(Control > Label)$_{WA}$	L	Amygdala / Parahippocampal Gyrus
	L	Parahippocampal Gyrus

Supplementary Table 2. H = Left (L), Right (R) cerebral hemisphere. Label = Emotion Label, Control = Control Text. CA = Chinese, WA = White American.

6 Supplementary Figures

Whole-brain Activation Results

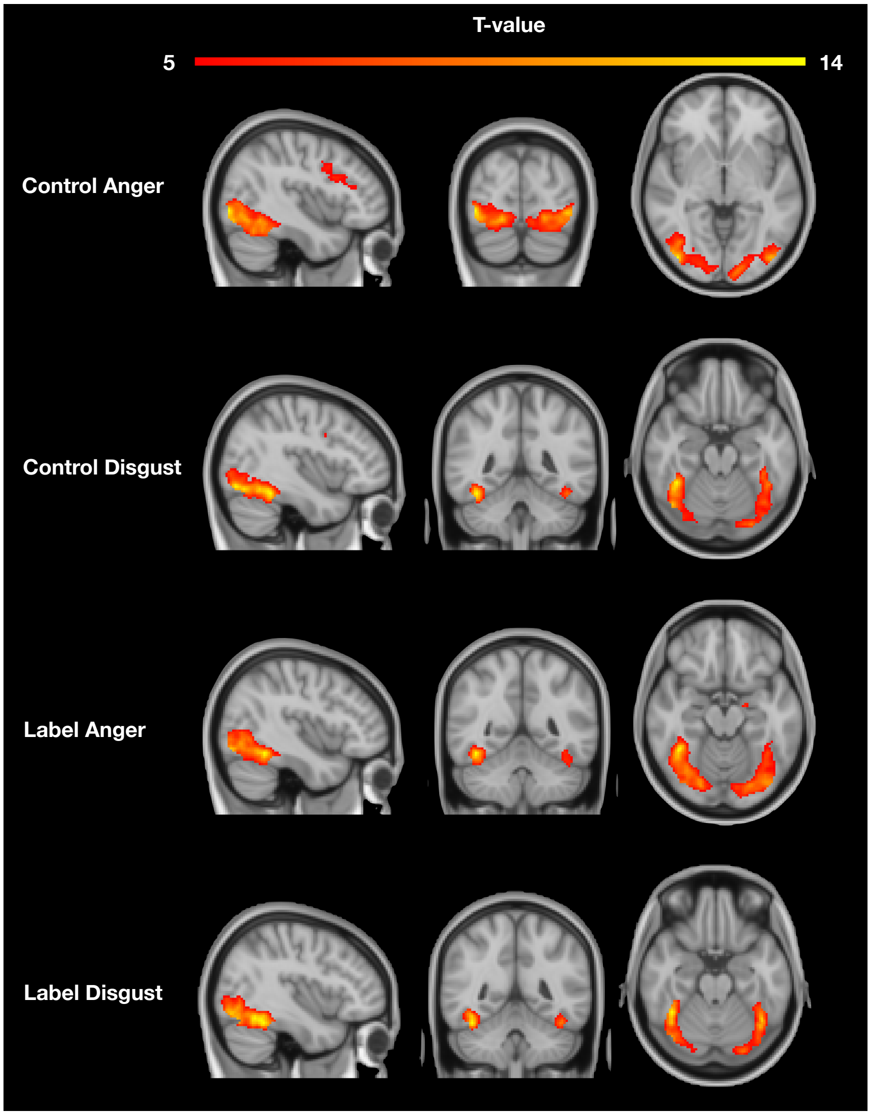

Supplementary Figure 1. Activation relative to baseline. Images centered at global maximum.

Regional Brain Activation Results

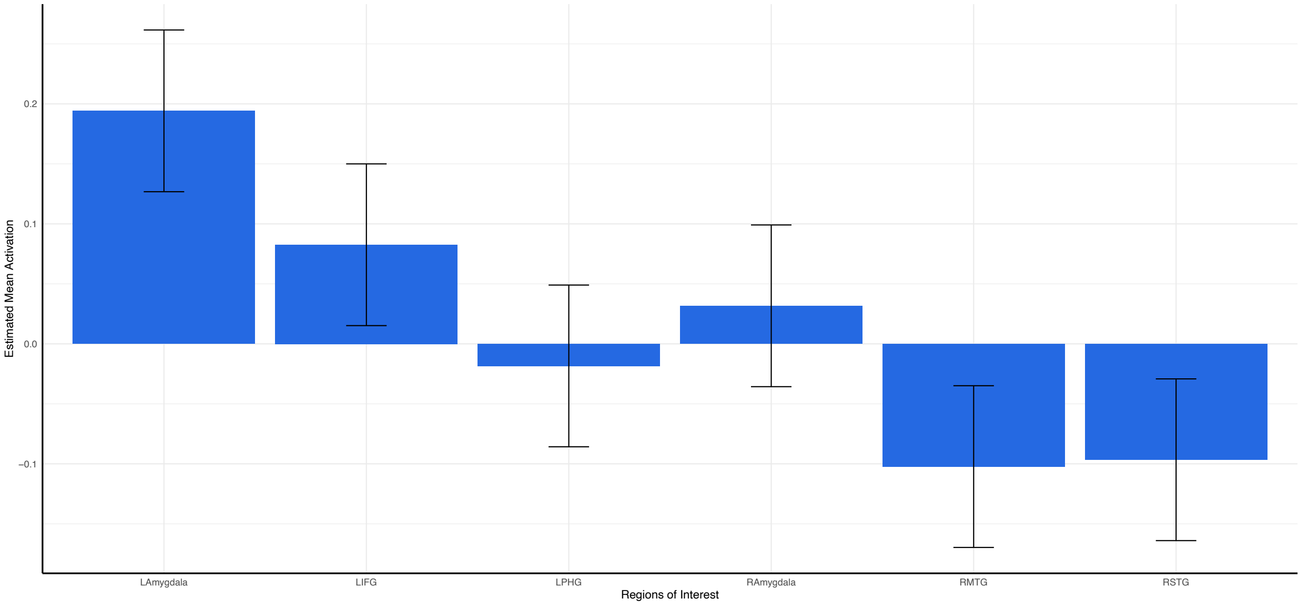

Supplementary Figure 2. Mean activation values across ROIs during emotion perception. Results are averaged over the levels of emotion category, prime type, cultural groups, and self-identified sex.

7 References

Barrett, L. F. (2006). Solving the Emotion Paradox: Categorization and the Experience of Emotion. *Personality and Social Psychology Review*, *10*(1), 20–46. https://doi.org/10.1207/s15327957pspr1001_2

Bates, D., Mächler, M., Bolker, B., & Walker, S. (2015). Fitting Linear Mixed-Effects Models Using lme4. *Journal of Statistical Software*, *67*(1). https://doi.org/10.18637/jss.v067.i01

Betz, N., Hoemann, K., & Barrett, L. F. (2019). Words are a context for mental inference. *Emotion*, *19*(8), 1463–1477. https://doi.org/10.1037/emo0000510

Brooks, J. A., Shablack, H., Gendron, M., Satpute, A. B., Parrish, M. H., & Lindquist, K. A. (2017). The role of language in the experience and perception of emotion: A neuroimaging meta-analysis. *Social Cognitive and Affective Neuroscience*, nsw121. https://doi.org/10.1093/scan/nsw121

Chiao, J. Y. (2018). Developmental aspects in cultural neuroscience. *Developmental Review*, *50*, 77–89. https://doi.org/10.1016/j.dr.2018.06.005

Gendron, M., Mesquita, B., & Barrett, L. F. (2020). The Brain as a Cultural Artifact: Concepts, Actions, and Experiences within the Human Affective Niche. In L. J. Kirmayer, C. M. Worthman, S. Kitayama, R. Lemelson, & C. Cummings (Eds.), *Culture, Mind, and Brain* (1st ed., pp. 188–222). Cambridge University Press. https://doi.org/10.1017/9781108695374.010

Kuznetsova A, Brockhoff PB, Christensen RHB (2017). lmerTest Package: Tests in Linear Mixed Effects Models. *Journal of Statistical Software*, *82*(13), 1-26. doi:10.18637/jss.v082.i13 https://doi.org/10.18637/jss.v082.i13

Lenth R (2022). emmeans: Estimated Marginal Means, aka Least-Squares Means. R package version 1.7.4-1, https://CRAN.R-project.org/package=emmeans

Lindquist, K. A., & Gendron, M. (2013). What's in a Word? Language Constructs Emotion Perception. *Emotion Review*, *5*(1), 66–71. https://doi.org/10.1177/1754073912451351

Lindquist, K. A., Jackson, J. C., Leshin, J., Satpute, A. B., & Gendron, M. (2022). The cultural evolution of emotion. *Nature Reviews Psychology*. https://doi.org/10.1038/s44159-022-00105-4

Lindquist, K. A., Satpute, A. B., & Gendron, M. (2015). Does Language Do More Than Communicate Emotion? *Current Directions in Psychological Science*, *24*(2), 99–108. https://doi.org/10.1177/0963721414553440

R Core Team (2022). R: A language and environment for statistical computing. R Foundation for Statistical Computing, Vienna, Austria. https://www.R-project.org/

Satpute, A. B., & Lindquist, K. A. (2019). The Default Mode Network's Role in Discrete Emotion. *Trends in Cognitive Sciences*, *23*(10), 851–864. https://doi.org/10.1016/j.tics.2019.07.003

Satpute, A. B., & Lindquist, K. A. (2021). At the Neural Intersection Between Language and Emotion. *Affective Science*, *2*(2), 207–220. https://doi.org/10.1007/s42761-021-00032-2

Wickham H, François R, Henry L, Müller K (2022). dplyr: A Grammar of Data Manipulation. R package version 1.0.9, https://CRAN.R-project.org/package=dplyr

Wickham H, Girlich M (2022). tidyr: Tidy Messy Data. R package version 1.2.0, https://CRAN.R-project.org/package=tidyr
